# Bacterial weaponry and the ecological factors of competitive success

**DOI:** 10.1042/EBC20250028

**Published:** 2026-09-02

**Authors:** Mia Gee, Connor Sharp

**Affiliations:** 1 School of Biosciences, Cardiff University, Cardiff, U.K.; 2 School of Biological Sciences, University of Reading, Reading, U.K.

**Keywords:** Bacteria, Bacterial warfare, Microbiome

## Abstract

Bacteria have evolved complex protein systems known as bacterial weapons to inhibit or kill their competitors. These bacterial weapons are a remarkably diverse arsenal that influence the composition and function of important microbial communities such as the human microbiome. In turn, the spatial constraints, nutrient availability, environmental stressors, and the presence of competitors determine not only whether weapons are expressed, but which weapons provide the greatest advantage. While bacterial weaponry is widespread, the types, mechanisms, and abundance of these systems vary between, and even within, species. Recent research has highlighted the importance of bacterial weaponry in community invasion and pathogenicity. Their potency and narrow killing spectrum have also generated interest in exploiting bacterial weapons to engineer microbial communities or develop therapeutics that avoid the disruption of broad-spectrum antibiotics. Understanding how ecological context affects weapon efficacy could reveal new virulence mechanisms used by pathogens and inform the design of novel treatments and microbiome-based therapies. This review outlines three of the best-studied bacterial weapon systems (protein bacteriocins, the type VI secretion system, and contact-dependent inhibition), highlighting their roles in microbial ecology, pathogenicity and their potential as therapeutics.

## Introduction

Bacteria live in dense and diverse communities where competition for resources is intense [1–4]. To succeed in competition, bacteria can employ different strategies, including exploitative competition, which relies on rapid nutrient acquisition [5,6], and interference competition, where specialised systems directly inhibit or kill rival cells. Among these diverse interference mechanisms are sophisticated peptide- and protein-based systems called bacterial weapons, that provide a powerful advantage in microbial conflicts, influencing who survives in a microbial community, with significant consequences for health and disease [7–11].

Weaponry is widespread in the bacterial kingdom, and a single bacterium can encode an arsenal of multiple mechanistically distinct weapons [12–16], from small molecules to large proteins and macromolecular complexes, such as diffusible protein bacteriocins (e.g., colicins and related toxins), contact-dependent inhibition (CDI) systems that require cell–cell contact, and the type VI secretion system (T6SS), a contractile nanomachine that delivers toxic effectors directly into target cells. Though these weapons span a broad mechanistic spectrum: they can deliver highly similar protein domains that kill target cells by attacking conserved targets.

Weaponry is common in bacteria, but the type and number of weapons can vary even between closely related species, suggesting that these weapons have evolved for competition within certain niches [13,17]. Weapons are often energetically costly, requiring the assembly of large macromolecular complexes [18] or, in extreme cases, death by cell lysis to release toxic proteins [17]. Consequently, their expression is often tightly regulated to ensure each system is only expressed under conditions where they are most effective. This regulation often involves sensing signals and environmental cues such as nutrient availability [19], temperature [20], pH [21], the presence of competitors [22,23], and the presence of clonemates [24,25] to ensure a coordinated and efficient attack.

The outcome of bacterial competition can have significant impacts on human health. Weapons can help pathogens overcome the colonisation resistance of the resident microbiota to establish infection [26,27] and bacterial weaponry is increasingly being associated with pathogens [11,13], while beneficial commensal strains can also rely on weaponry to persist within the human microbiome [28,29]. Beyond their impact on ecology, bacterial weapons are often potent, specific antimicrobials with activity against some of the most important antimicrobial-resistant pathogens. With the rise of antimicrobial resistance (AMR) across the globe and the scarcity of new drugs to fight infections, there has been renewed interest in their potential in the next generation of antibiotics or probiotics [30–32].

While bacteria can compete using a variety of small metabolites such as antibiotics and antimicrobial peptides, which have been extensively reviewed elsewhere [33,34], this review focuses on large protein bacterial weapons, exploring the biochemistry and ecological role of protein bacteriocins, T6SS and CDI (Table 1).

**Table 1:** Ecological, genetic, and structural differences between three major bacterial weaponry systems

Weapon	Contact	Genetic Structure	Size	Spectrum
Protein bacteriocins	Independent	Toxin and downstream immunity followed by a lysis gene in some cases.	∼29–70 kDa [42]	Found mostly within Gammaproteobacteria [36] with a narrow spectrum of activity against strains closely related to the producer.
Type VI secretion system (T6SS)	Dependent	13 core components with additional structural genes and a repertoire of secreted effectors [85]	Membrane complex of ∼1.7 MDa [120] and a needle with length of ∼750 nm [121]	Broad antibacterial spectrum against Gram-negative bacteria with fewer known Gram-positive effectors [122]. Can also deliver anti-eukaryotic and nutrient-scavenging effectors.
Contact-dependant inhibition (CDI)	Dependent	Toxin and downstream immunity. Can also encode an orphan gene of unknown function. Outer membrane secretion partner can be either upstream of the toxin or downstream of the immunity gene.	Variable length toxin ∼180–630 kDa. Can protrude 33 nm from the cell membrane [109]	Widespread in Proteobacteria with a narrow spectrum of activity against strains closely related to the producer [107].

### Protein bacteriocins

First identified in 1925, bacteriocins were one of the earliest bacterial weapons to be discovered, when a culture of *Escherichia coli* was found to inhibit a neighbouring strain by a secreted factor [35]. Since then, they have been found throughout bacteria [36–38], and the term bacteriocins has developed to cover a diverse range of peptides and proteins secreted by bacteria to inhibit or kill target bacteria. Though multiple classification systems exist [39], they are commonly grouped into small peptide bacteriocins (Class I and II) [38] and large protein bacteriocins, which are only found in Gram-negative bacteria (Class III). Bacteriocins from Gram-positive bacteria and small peptide bacteriocins include important bacteriocins such as nisin [40], a recognised food preservative, and have been examined elsewhere [38,39,41]. Here, we discuss the Class III large protein bacteriocins, which also have significant impacts on bacterial ecology and have distinctive potential as future therapeutics.

### Structure and function

Unlike peptide bacteriocins, which can be <5 kDa, protein bacteriocins are large, multi-domain proteins typically 40–70 kDa. They tend to have potent activity but a narrow spectrum of activity, targeting only closely related organisms to the producer [42,43]. The most well-studied are the colicins, which specifically target *E. coli* [42], though homologues can be found in multiple pathogens, including *Pseudomonas aeruginosa* (pyocins) [44], *Klebsiella pneumoniae* (klebicins) [45], *Salmonella enterica* (salmocins) [46], and *Burkholderia spp*. (burkicins) [47]. However, so far, they appear to be restricted to the gamma- and beta*-proteobacteria* [36,47]. They are encoded as a single gene for the toxin with N-terminal domains involved in receptor binding and translocation and a C-terminal cytotoxic domain, followed immediately downstream by an immunity protein that prevents cell death in the producer [42]. This can then be followed by a lysis gene responsible for lysing the cell, causing release of the toxin [42].

**Figure 1:**
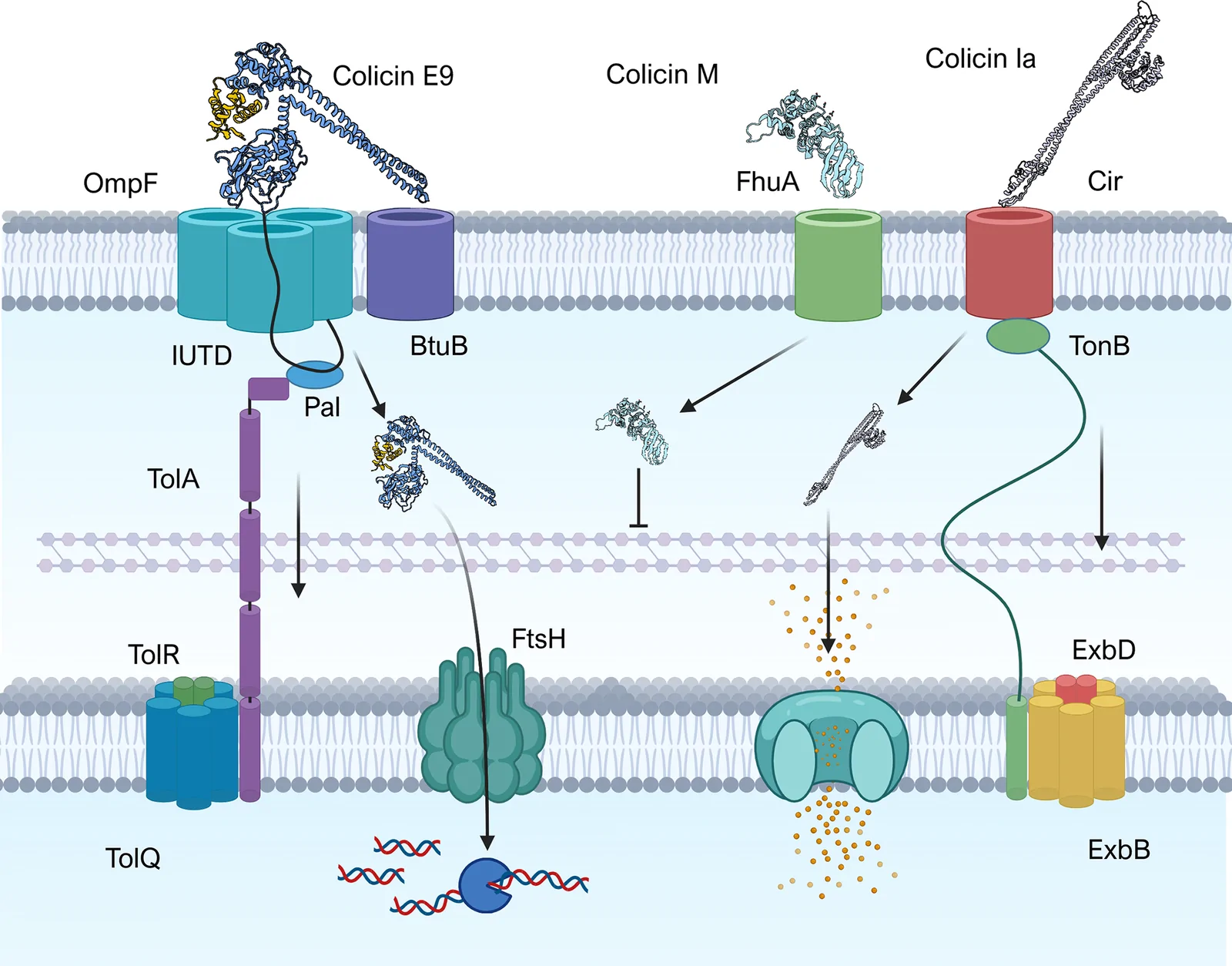
Protein bacteriocins hijack multiple proteins in the target bacteria to pass through membranes To pass the outer membrane, protein bacteriocins such as colicins hijack outer membrane receptors involved in nutrient import. After binding to the receptor, colicins can either thread an intrinsically unstructured translocation domain (IUTD) through a separate translocator protein (OmpF as shown for Colicin E9) or translocate through the receptor protein (FhuA or Cir for Colicin M and Colicin Ia, respectively). The energy for passing through the OM is provided by interactions with PMF coupled complexes Tol (sometimes via Pal) and Ton. Retraction of TolA and TonB drags unfolded colicins through the OM into the periplasm, where they are thought to refold. Colicins can then exert their cytotoxic activity by either degrading peptidoglycan precursors (Colicin M), forming a pore in the inner membrane (Colicin Ia) or passing through the inner membrane by a charge-dependent interaction with lipids and FtsH to degrade nucleic acids (Colicin E9). Created in BioRender. Sharp, C. (2026) https://BioRender.com/50txouz.

For colicin-like proteins to kill a target cell, they must first pass through the Gram-negative outer membrane (OM) (Figure 1). This presents an interesting question of how a 70 kDa protein is able to pass through the formidable barrier of the Gram-negative bacteria, which is capable of blocking the passage of many antibiotics. To translocate across the OM, colicins first hijack OM receptors, proteins involved in the import of different nutrients. After binding receptors, colicins interact with either the Ton or Tol system, proton motive force-coupled complexes that are able to physically pull the colicin through the OM [48]. Once through, colicins can inhibit or kill either by degrading lipid II (ColM, PaeM1/4, BurkM), forming a pore in the inner membrane disrupting the ion gradient (ColE1, PyoS5), or passing through the inner membrane by an unclear mechanism involving a bacteriocin domain also found in certain T6SS toxins [49] and interactions with inner membrane lipids [50], and inner membrane proteins [51]. Once through the inner membrane, protein bacteriocins can degrade DNA (ColE9, PyoS2, KlebB), tRNA (ColD, ColE5, PyoS4) or rRNA (ColE3, CloDF13, PyoS6) [12,42,52].

### Ecological impact of protein bacteriocins

Though bacterial weapons have huge potential to impact the composition of bacterial communities, the role of bacteriocins in ecology has been the subject of controversy. Studies often find conflicting results, ranging from bacteriocins having an important role in determining community composition [26,29,53], to having little effect [54–56], to even suggesting that bacteriocins have no impact on a community and only act as selfish genetic elements to maintain themselves in the genome, similar to a traditional toxin–antitoxin system [57,58]. But why are there so many conflicting results? As colicin-like proteins rely on binding a receptor protein on the OM of the target bacteria, their efficacy is highly influenced by the abundance of their receptors [8]. These receptors are mostly proteins involved in the import of important nutrients such as nucleosides (Tsx [59]), sugars (OmpF [60]), cobalamin (BtuB [61]), or iron and ferri-siderophores (FhuA [62], Cir [63], FepA [64], FpvA [65]). The abundance of these receptors in the OM of target bacteria is influenced by multiple environmental factors, such as the abundance of the nutrients they import [66,67], pH [68], and osmolarity [69]. The local environment can therefore impact the efficacy of colicin-like proteins, with certain bacteriocins more effective under different environmental conditions. For instance, Colicin Ib, which targets the siderophore receptor Cir, has been shown to be important for competition between *E. coli* and *Salmonella* but only during inflammation [8], where the host limits the available free iron, which causes an up-regulation of siderophore receptors. In turn, many bacteriocins which target siderophore receptors are regulated by Fur, ensuring maximal bacteriocin expression during low-iron conditions where they are most effective [13,70].

### Regulation of protein bacteriocins

Colicin-like proteins have no typical secretion system for their export. Instead, they are released by lysis and cell death, either by expressing a dedicated lysis protein or by co-opting the lysis genes of phages [71,72]. This high cost of their release, combined with the influence of the chemical and spatial environment [73] on their efficacy, means colicins are tightly regulated, often with multiple regulatory elements combining to ensure expression only under optimum conditions, including Fur, CsrA [19], and the SOS response, sensing damage from colicin-producing clonemates and competitors [74,75].

The conditional dependence of colicin regulation and efficacy suggests that colicins may have evolved to allow bacteria to compete and benefit in certain niches. Recent studies of uropathogenic *E. coli* (UPEC) have identified links between these iron-uptake receptor-targeting colicins and UPEC pathogenicity, suggesting that these bacteriocins enable *E. coli* to survive in the human microbiome for longer periods of time, outcompeting commensal strains and allowing them to spread to other niches where they can cause disease, such as the urinary tract and bloodstream [11,36,76]. Though the exact role colicins play in pathogenicity is still being uncovered, as we learn more about the interactions of pathogens with bacterial communities such as the microbiome, they could play a significant role in disease and human health.

### Colicin-like proteins as novel antimicrobials

The killing activity of colicin-like proteins, able to kill down to the picomolar level, combined with their narrow spectrum of activity makes them promising antimicrobials for removing pathogens from the microbiome without causing dysbiosis [30]. The modular domain structure of colicin-like proteins allows new combinations of receptor targeting and killing activity to be generated to expand killing spectrum or overcome natural resistance [30]. They have been shown to be effective antimicrobials against important pathogens responsible for AMR-related deaths, including *E. coli* [77], *K. pneumoniae* [45], and *P. aeruginosa* [78].

## The type VI secretion system

**Figure 2:**
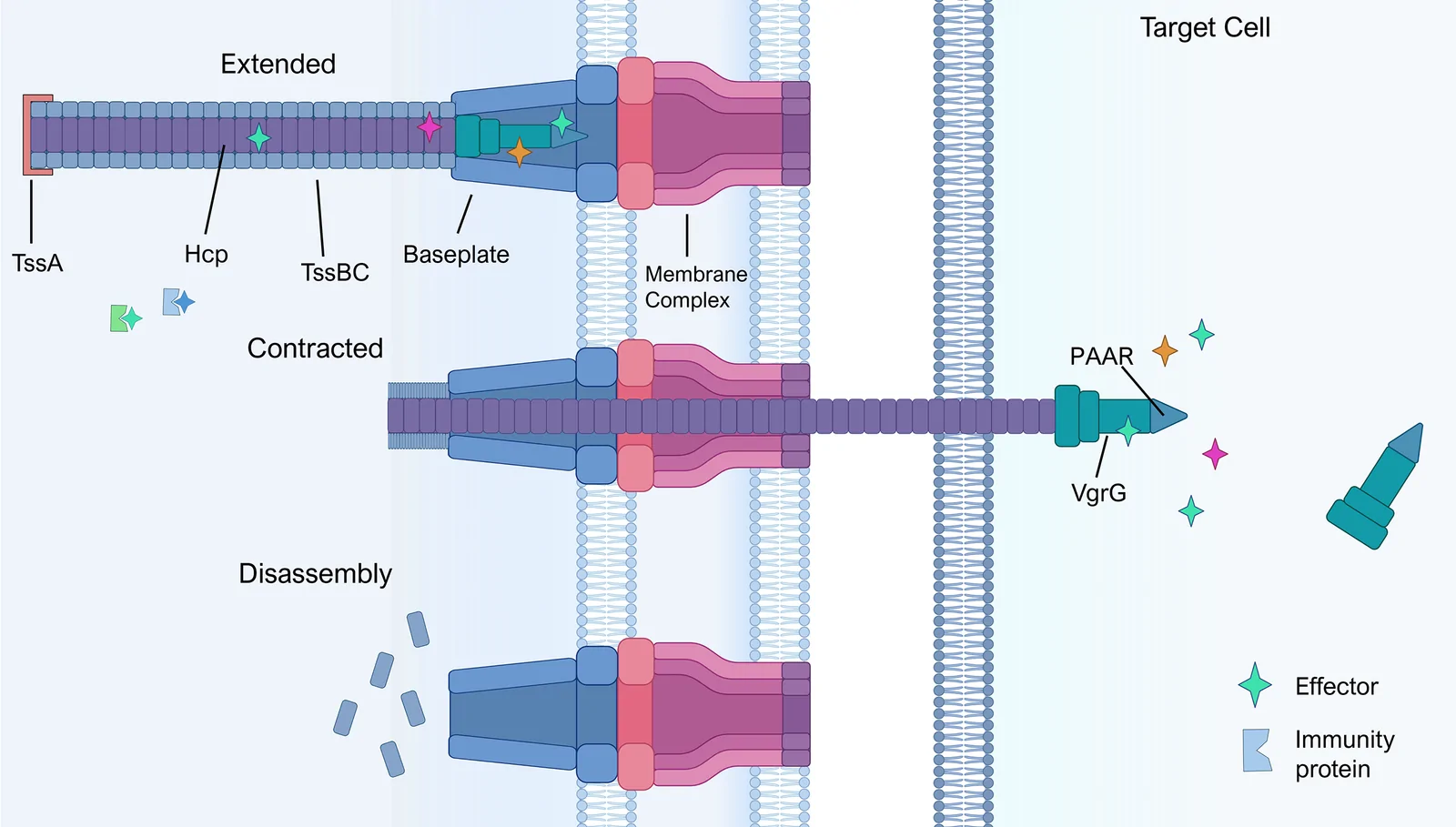
Structure and firing of the T6SS The membrane complex, constructed from TssJ, TssL, and TssM proteins, is the first to assemble on the cell envelope and forms a pore structure connecting the cytoplasmic proteins to the external environment. Once bound, the membrane complex facilitates the recruitment of the baseplate containing (TssE, TssF, TssG, TssK). This structure holds the tip of the spear, the spike complex, VgrG and PAAR. A trimer of VgrG forms a conical shape, thought to help pierce the membrane, at the top of the polymer channel. Located at the cone vortex, PAAR (proline–alanine–alanine–arginine) sharpens the tip via zinc-coordinated tightening of the structure. TssA acts as a cap on the distal end of the inner and outer sheath (the stem-channel polymers). TssA coordinates the inner tube, Hcp (haemolysin co-regulated protein), and the outer sheath's, TssB and TssC, polymerisation until they reach the other end of the cell (extended). After receiving a signal, the baseplate undergoes a conformational change, which rapidly contracts the sheath of the structure (contracted), which ejects the inner tube with its attached spike complex out of the cell into the extracellular environment or target bacterial or eukaryotic cell. This process is recyclable. ClpV, also referred to as TssH, an ATPase, separates the components of the contracted sheath and returns them to the cytoplasm for use in subsequent firing (disassembly). Effector proteins can be loaded onto the T6SS apparatus via interactions with the Hcp, VgrG or PAAR proteins by covalent or noncovalent bonds. The mechanism and structure of T6SS have been reviewed elsewhere [123]. Created in BioRender. Sharp, C. (2026) https://BioRender.com/be6lzjk.

T6SS is a nanomolecular complex that translocates proteins and effectors to enhance the survival and competitiveness of encoding bacterial species across diverse ecological niches [79]. The T6SS is commonly compared with the contractile tail of T4 bacteriophage due to its structurally homologous components and similar method of assembly [80]. Functionally, T6SS acts as a molecular harpoon, using a rapidly contracting sheath to propel effectors into neighbouring cells or into the extracellular environment (Figure 2). Therefore, unlike the protein bacteriocins, the T6SS requires direct contact with the target cell.

Widely encoded among Gram-negative bacteria, with approximately 25% of species containing at least one T6SS structural cluster, the T6SS exhibits evolutionary and structural diversity [17,81]. Though most subtypes share a conserved set of core components, comparative genomic analyses show variation in gene-cluster architecture and sequence diversity [81]. Based on these factors, T6SS can be divided into four main subtypes, T6SSi-iv. The most comprehensively studied is T6SSi, predominantly found in *Proteobacteria*; it encompasses a wide range of systems and is divided into six subgroups (1, 2, 3, 4a, 4b, and 5) [81]. Of the remaining T6SS subtypes: T6SSii is largely restricted to the *Francisella species* [82], T6SSiii is found in the *Bacteroidetes* phylum [83], and the proposed fourth form, T6SSiv, is found in *Amoebophilus asiaticus* [84].

An individual bacterium can encode multiple T6SS clusters, each with their own repertoire of effectors [85]. Effector proteins are attached to the T6SS apparatus by either covalently or non-covalently binding proteins in the tube (Hcp [86]) or spike (VgrG [87], PAAR [88]), and a T6SS cluster can deliver multiple effectors simultaneously. Even amongst strains of the same species, there can be staggering diversity in the effector proteins deployed by the T6SS. For example, a survey of nearly 2000 *P. aeruginosa* genomes found 335 distinct effector repertoires, with 30% of effectors present in less than 25% of strains [15,85]. The huge diversity of effectors means that even within a single bacterium, the T6SS is capable of having multiple overlapping effects on hosts, bacteria and non-bacterial microbes such as amoeba.

### Ecological impact of protein bacteriocins

#### T6SS in bacterial warfare

T6SS interbacterial effectors can target many of the same cellular structures as protein bacteriocins, such as DNA (Tde2, RhsAB), peptidoglycan (Tae1-4, TaeX) and the inner membrane (Tse4, VasX), and these effectors can share high similarity with the cytotoxic domains of protein bacteriocins [89]. As with protein bacteriocins, these effectors are often encoded as toxins followed by immunity proteins which prevent death of the producing cell. Interestingly, as a result of the multiple different effectors deployed simultaneously, T6SS resistance is less likely to arise through mutation or horizontal transfer of a single immunity gene [90]. Though T6SS systems encode diverse effectors, they are enriched in effectors that cause lysis, as this prevents the formation of a barrier of dead cells, increasing the efficacy of the T6SS [91]. Effectors that target the host can also influence the composition of microbial communities. Effectors can prevent phagocytosis or promote internalisation by non-phagocytic cells to provide a replicative niche [92]. Other effectors can interfere with the host’s innate immunity to benefit the producing bacteria by stimulating inflammation [93] or trigger host expulsion of competing bacteria [94]. T6SS effectors can also engage in exploitative competition by secreting factors which bind nutrients such as zinc [95] and iron [96] in the environment, sequestering them away from competitors.

The T6SS has been shown to provide bacteria with fitness benefits in a variety of different environments, though with the overlapping roles of different effectors it can be difficult to determine if this is due to host or interbacterial effects. Within the gut microbiome, there are an estimated 10^9^ T6SS first events per gram of faecal matter per minute in a gnotobiotic mouse [97], highlighting the potential impact of T6SS on bacteria in this niche. The T6SS is found in many enteric pathogens, and its role as an interbacterial weapon (primarily against *Enterobacteriaceae*) has been demonstrated to support gut colonisation of pathogens such as *Shigella sonnei* [98], *Salmonella Typhimurium* [99], and *K. pneumoniae* [100]. But it is not only pathogens that use T6SS. Commensal bacteria can also encode T6SSs and *B. fragilis* can limit the colonisation of enterotoxic strains in a T6SS-dependent manner [10].

#### Regulation of the T6SS

The multiple T6SSs encoded per strain and the diverse ways that T6SS can interact with the environment have led to complex regulation of T6SS, with a wide variety of known regulators. T6SS can respond to environmental cues such as oxygen [101], nutrients [102], temperature [20], and host molecules such as bile salts and mucin [103]. T6SSs can also respond to cell density via quorum sensing [24] and can even respond to an attack, with cells building and firing a T6SS in the direction of the competitor’s T6SS attack in a strategy known as tit-for-tat [104]. T6SS regulation can also differ between closely related strains; for instance, *Vibro parahemolyticus* T6SS is up-regulated in warmer temperatures [105], whilst *V. cholerae* activity is up-regulated in colder temperatures [20]. Even T6SS clusters within the same bacterium are differentially regulated. In *P. aeruginosa*, H1-T6SS is responsible for interbacterial combat and is up-regulated under low cell density, whereas under high cell density, H2 and H3 T6SS systems are up-regulated [24]. Though T6SS expression does not require the cell sacrifice of protein bacteriocins, and the exact cost of T6SS production and firing is predicted to be small to none [106], like the bacteriocins, the complex and diverse regulatory systems likely ensure that the correct T6SS system is expressed under the optimum conditions.

Ultimately, the diverse array of T6SS and effectors allows bacteria to compete and manipulate their environment to survive. The ability of T6SS to aid or prevent colonisation suggests that they could have a role in engineered probiotics, however, harnessing them is likely to be more complex than deploying protein bacteriocins, which can be delivered like conventional antimicrobial agents.

## Contact-dependent inhibition

Like the T6SS, CDI is widely distributed across Proteobacteria, including many pathogens [107], and also requires contact between cells. Unlike the T6SS, however, CDIs also hijack multiple protein receptors to deliver a C-terminal toxin domain into the target cell. Though they share many similarities with both T6SS and protein bacteriocins, and can deliver similar toxin domains, CDIs have a unique structure and mechanism of delivery.

**Figure 3:**
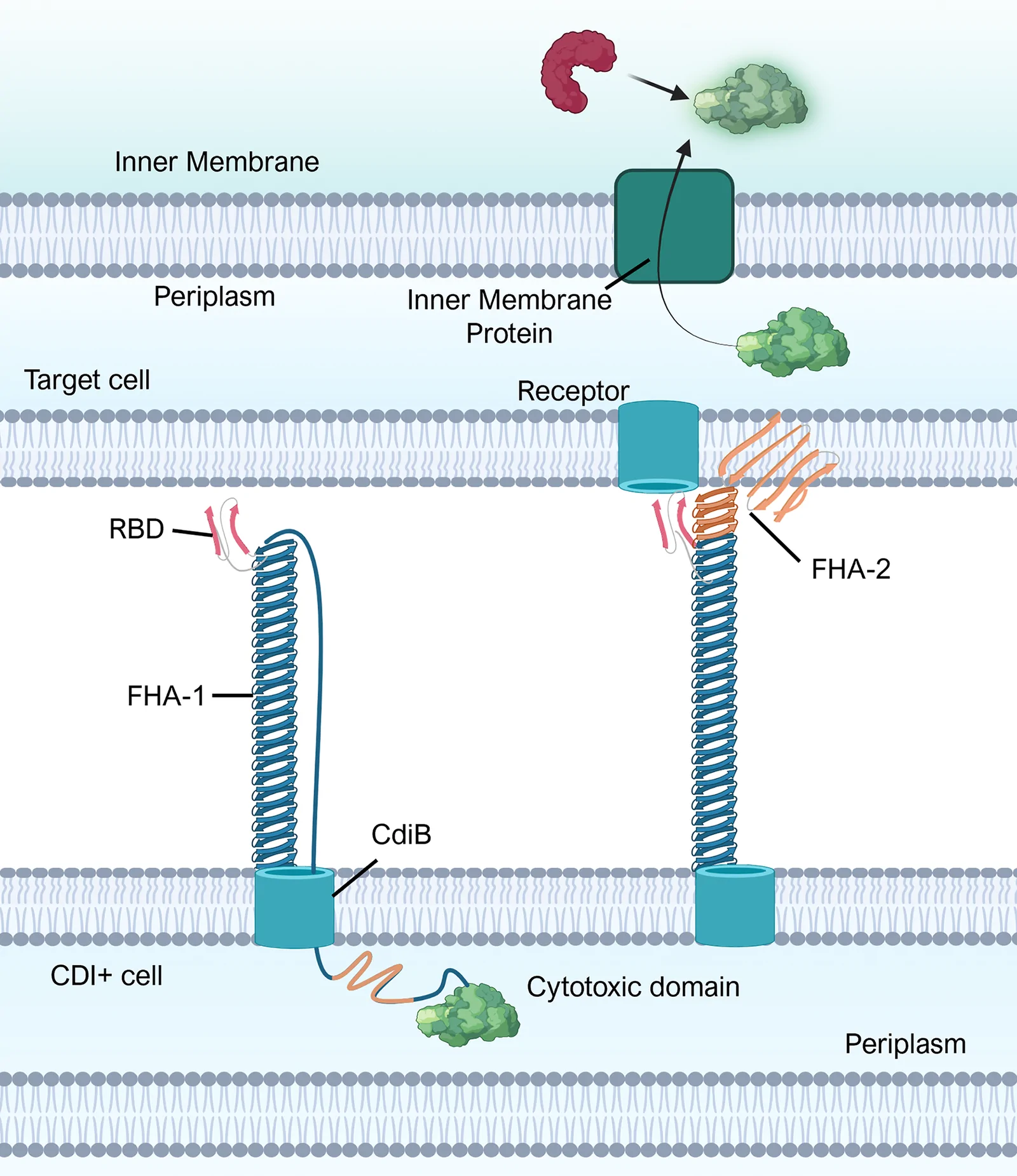
CDI structure and delivery mechanism CdiB transports an unfolded CdiA protein through the OM. It is thought that the folding of CdiA β-helix drives the export of CdiA. At some point, the transport is halted by a secretion arrest domain in CdiA, which maintains the cytotoxin C-terminal domain in the periplasm and leaves the receptor-binding domain at the tip of CdiA filament. Upon binding of the receptor, secretion is triggered through an unknown mechanism. FHA-2 domain then allows passage through the OM. As the cytotoxic domain passes through the OM into the periplasm, it is cleaved from the rest of CdiA at a specific site called the VENN motif. The cytotoxic domain then interacts with an inner membrane protein to facilitate translocation across the inner membrane. CdiA cytotoxic domains can then target nucleic acids, the inner membrane or other cell targets [109]. Cytotoxic domains may need to interact with cytoplasmic proteins in the target cell to activate. Created in BioRender. Sharp, C. (2026) https://BioRender.com/4k00l5q.

CDIs are encoded as a two-partner system with an outer membrane transporter (CdiB) that exports a large (sometimes >700 kDa) protein (CdiA) with a C-terminal toxin domain [107]. Like colicins and T6SS effectors, the toxin is followed by downstream immunity proteins, which prevents death of the producer. CdiA forms a long filament that extends ∼33 nm out of the membrane, however, they have a more complex structure and mechanism than the original model of a ‘toxin on a stick’ [108]. The toxin C-terminus is actually held in the periplasm until the receptor binding domain, at the midpoint of CdiA, binds to an outer membrane receptor in the target cell (Figure 3). Like bacteriocins, these receptors can be nutrient importers or BamA of the Bam complex, a conserved protein responsible for assembling β-barrel proteins in the OM. Once bound, the C-terminal domain is released from the periplasm where it can pass through the target OM without using a receptor [109]. Once through the OM, the toxin is cleaved and passes through the inner membrane through an interaction with different integral membrane proteins [109]. CdiA can encode a wide range of diverse toxins, even with strains of the same species using different toxins, including nucleases, NADH glycohydrolases, cytidine deaminases, and ionophores [107,110]. However, unlike protein bacteriocin toxins, which are active once they pass the inner membrane, many CDI toxins require activation by a protein in the target cell such as CysK [42] or EuTF [111].

### Regulation and ecological impact of CDI

Regulation of CDI genes appears to differ even within the same species. The first CDI system identified, CdiAB in *E. coli* EC93, expresses the system constitutively [112], whereas another *E. coli* CDI system in UPEC 536 is not expressed at all under lab conditions [107], and *Burkholderia thailandensis* CDI homologues are regulated by quorum sensing [113]. CDI activity is also influenced by the environment. Efficacy of CDI systems increased 10,000-fold with bacterial motility compared with non-motile scenarios, as motility allows CDI^+^ bacteria to interact with more target bacteria [114]. CDI is also more effective when the attacking strain is outnumbered and allows strains at low frequency to invade a community, in contrast to protein bacteriocins which require higher cell density to effectively compete [115]. While the competitive benefit of CDI has been demonstrated in many species, CDI can also increase cooperation between CDI^+^ strains. Expression of CDI increases adhesion between strains and biofilm formation in strains which possess the same CDI^+^ and receptors [116,117]. As CDI targets only closely related strains, this signalling allows strains to induce cooperative behaviours in closely related strains or clonemates [116,117]. CDI may therefore have a dual role in pathogenicity, killing CDI^-^-competitors while also signalling CDI^+^-clonemates and coordinating cooperative behaviours such as biofilm formation.

There has been limited study of the theraputic antibacterial potential of CDI. Like the T6SS, CDI-based interventions would require delivery via a live probiotic chassis, but their reliance on protein receptors for import gives them a narrow killing spectrum. As CDI systems are widespread among pathogenic bacteria, targeting or disrupting CDI-mediated interactions could offer a promising strategy to weaken pathogens and reduce their capacity to cause disease [118,119].

## Conclusion

Bacteria can deploy a wide range of weaponry to compete with their rivals. Though these systems use diverse delivery mechanisms, they can deliver very similar toxins. While many strains have an arsenal of weaponry, they are often tightly regulated to ensure the best weapon is deployed for competition in that niche. Weaponry is widespread across clinically important pathogens and is increasingly linked to the ability of strains to displace commensals within the microbiota. Given the remarkable diversity of bacterial weapons and the equally varied ecological niches in which bacteria compete, an important challenge is to understand how cells select, coordinate, and optimise their arsenal of weapons for different environments. Such insights could help identify strategies to prevent pathogens persisting in the microbiome, engineer resilient microbial communities, and ultimately harness bacterial weaponry as targeted antimicrobials to combat pathogens.

## Summary

- Bacterial weaponry is widespread across bacteria and influences microbial community composition.
- Weapons are structurally and mechanistically diverse but also share some conserved features and cellular targets.
- Weaponry is often tightly regulated and different weapons are expressed for the specific environment.
- Pathogens can use weaponry to invade a bacterial community, with significant implications for health and disease.

## Open Access

Open access for this article was enabled by the participation of the University of Reading in an all-inclusive Read & Publish agreement with Portland Press and the Biochemical Society under an agreement with JISC.

## Abbreviations

AMR: antimicrobial resistance
CDI: contact-dependent inhibition
Hcp: haemolysin co-regulated protein
OM: outer membrane
PAAR: proline–alanine–alanine–arginine
T6SS: type VI secretion system
UPEC: uropathogenic *Escherichia coli*
